# Amphiphilic Thermoresponsive Triblock PLA-PEG-PLA and Diblock mPEG-PLA Copolymers for Controlled Deferoxamine Delivery

**DOI:** 10.3390/gels11090742

**Published:** 2025-09-15

**Authors:** Nikolaos D. Bikiaris, Ermioni Malini, Evi Christodoulou, Panagiotis A. Klonos, Apostolos Kyritsis, Apostolos Galaris, Kostas Pantopoulos

**Affiliations:** 1 Laboratory of Polymer Chemistry and Technology, Department of Chemistry, Aristotle University of Thessaloniki, 54124 Thessaloniki, Greece; ermioni18@gmail.com (E.M.); pklonos@central.ntua.gr (P.A.K.); 2 Dielectrics Research Group, Department of Physics, National Technical University of Athens, Zografou Campus, 15780 Athens, Greece; akyrits@central.ntua.gr; 3 Lady Davis Institute for Medical Research Montreal, and Department of Medicine, McGill University, Montreal, QC H3T 1E2, Canada; apostolos.galaris@mail.mcgill.ca (A.G.); kostas.pantopoulos@mcgill.ca (K.P.)

**Keywords:** thermoresponsive polymers, poly(lactic acid), poly(ethylene glycol), micelles, hydrogel, emulsions, deferoxamine, controlled drug release, thalassemia

## Abstract

This study focuses on the synthesis and characterization of thermoresponsive hydrogels of poly(lactic acid) (PLA) and poly(ethylene glycol) (PEG), PLA–PEG copolymers, aiming at the targeted and controlled release of deferoxamine (DFO), a clinically applied iron-chelating drug. Triblock (PLA-PEG-PLA) and diblock (mPEG-PLA) copolymers were synthesized using ring-opening polymerization (ROP) with five different PEGs with molecular weights of 1000, 1500, 2000, 4000, and 6000 g/mol and two types of lactide (L-lactide and D-lactide). Emulsions of the polymers in phosphate-buffered saline (PBS) were prepared at concentrations ranging from 10% to 50% *w*/*w* to study the sol–gel transition properties of the copolymers. Amongst the synthesized copolymers, only those that demonstrated thermoresponsive sol-to-gel transitions near physiological temperature (37 °C) were selected for further analysis. Structural and molecular confirmation was performed by Nuclear Magnetic Resonance (NMR) and Fourier-transform infrared spectroscopy (FTIR), while the molecular weights were determined via Gel Permeation Chromatography (GPC). The thermal transitions were studied by calorimetry (DSC) and crystallinity via X-ray diffraction (XRD) analysis. DFO-loaded hydrogels were prepared, and their drug release profiles were investigated under simulated physiological conditions (37 °C) for seven days using HPLC analysis. The thermoresponsive characteristics of these systems can offer a promising strategy for injectable drug delivery applications, where micelles serve as drug carriers and undergo in situ gelation, enabling controlled release. This alternative procedure may significantly improve the bioavailability of DFO and enhance patient compliance by addressing key limitations of conventional administration routes.

## 1. Introduction

Thalassemias are genetic blood disorders characterized by reduced or absent synthesis of α- or β-globin chains in hemoglobin, leading to ineffective erythropoiesis, chronic anemia, and iron overload due to frequent blood transfusions [1]. Deferoxamine (DFO), an iron chelating drug, is widely used for managing systemic iron accumulation in patients with transfusion-dependent thalassemia [2]. Due to poor oral bioavailability and low plasma half-life (~20 min), DFO is typically administered by subcutaneous overnight injections with the aid of an infusion pump at doses around 40–60 mg/per kg of body weight per day, at least 5 days per week [3]. However, this cumbersome procedure reduces patient compliance and increases the risk of complications.

To address these challenges, advanced drug delivery systems based on biocompatible and biodegradable polymers have gained significant attention. Over the past decades, polymer-based systems have attracted increasing interest in biomedical applications due to their capacity for controlled release [4]. Poly(lactic acid) (PLA), an aliphatic polyester derived from renewable resources [5], has been widely explored for drug delivery [6] thanks to its biocompatibility and biodegradability [7]. Nonetheless, PLA exhibits hydrophobicity and relatively slow degradation, characteristics that limit its standalone application in sustained release of hydrophilic drugs such as DFO [8].

Amphiphilic block copolymers composed of PLA and poly(ethylene glycol) (PEG) exhibit promising properties for drug encapsulation and controlled release. PLA provides hydrophobic domains for drug loading, while PEG contributes hydrophilicity, enhancing drug solubility and reducing immunogenicity [9]. These polymers can self-assemble into micelles or undergo sol–gel transitions in response to temperature changes, forming thermoresponsive hydrogels suitable for injectable formulations. Thermoresponsive polymers are materials that display reversible phase transitions following temperature changes, typically near physiological conditions [10]. These polymers can shift from a sol (liquid) to a gel (semi-solid) state upon heating, enabling minimally invasive administration and in situ gel formation. This property makes them highly desirable for drug delivery applications, as they allow for localized, sustained, and controlled drug release [11]. Abebe and Fujiwara [12] demonstrated the utility of stereocomplexed PLA–PEG–PLA hydrogels in controlled release applications, while Movaffagh et al. [13] investigated in situ forming thermoresponsive hydrogels for levothyroxine sodium with promising in vitro release behavior.

Currently, no DFO-based hydrogels have yet reached the market; however, other thermoresponsive hydrogel-based formulations are commercially available. Formulations like Pluronic^®^ F127 (Poloxamer 407) exemplify this utility, serving in various topical and injectable applications where the gelation at body temperature facilitates controlled drug delivery with improved patient compliance and reduced systemic side effects [14]. Another notable example is Atridox^®^, a doxycycline hyclate-loaded resorbable polymer gel used in periodontal therapy, which demonstrates the clinical translation of thermoresponsive hydrogels to provide targeted, long-lasting antibiotic delivery directly to periodontal pockets [15].

Consequently, in the current work, thermoresponsive PLA–PEG copolymers were synthesized and evaluated as injectable carriers for the targeted delivery of DFO. Both triblock (PLA–PEG–PLA) and diblock (mPEG–PLA) copolymers were prepared, moreover, with varying PEG molecular weights and stereoisomeric lactide forms (PLLA and PDLA). Various molecular weights of PEG were used to examine the effect of molecular weight on the hydrophilicity, crystallinity, gelation temperature, and thermoresponsive behavior of the polymeric systems, while two lactide stereoisomers were also used to explore any potential stereocomplex effects on gelation and release. Their thermoresponsive behavior and ability to encapsulate and release DFO, under conditions analogous to those of the human body, were investigated. While earlier studies employed PLA–PEG–PLA copolymers mainly for hydrophobic or moderately soluble drugs, our work specifically targets deferoxamine, a clinically challenging, highly hydrophilic iron chelator with poor bioavailability. In addition, our study uniquely combines in vitro release kinetics with bioactivity validation of released DFO in cultured cells, providing dual functional confirmation. This extends the scope of PLA–PEG copolymer hydrogels beyond previous applications and establishes their potential for clinically relevant DFO delivery. The developed systems aim to improve DFO delivery by forming micelles in solution that transition into a gel upon injection, providing sustained release and enhanced therapeutic efficiency.

Unlike prior studies that primarily focused on release kinetics, our study combines thermoresponsive PLA–PEG copolymer design with dual assessment of deferoxamine release and biological activity in human cells. This comprehensive evaluation demonstrates not only controlled release but also retained bioactivity, providing an advance over previously published systems. Building upon these approaches, our study introduces a novel thermoresponsive PLA–PEG-based delivery platform specifically tailored for DFO, with a comprehensive evaluation of in vitro drug release and drug efficacy in a cell culture model. Thus, the novelty of this study lies in combining a broader compositional design with a clinically relevant hydrophilic drug and validating not only physicochemical release but also drug functionality in a biological context. This dual assessment highlights the potential for sustained therapeutic action, reduced dosing frequency, and improved patient compliance in the treatment of β-thalassemia.

## 2. Results and Discussion

### 2.1. Characterization of Synthesized Copolymers

**Figure 1:**
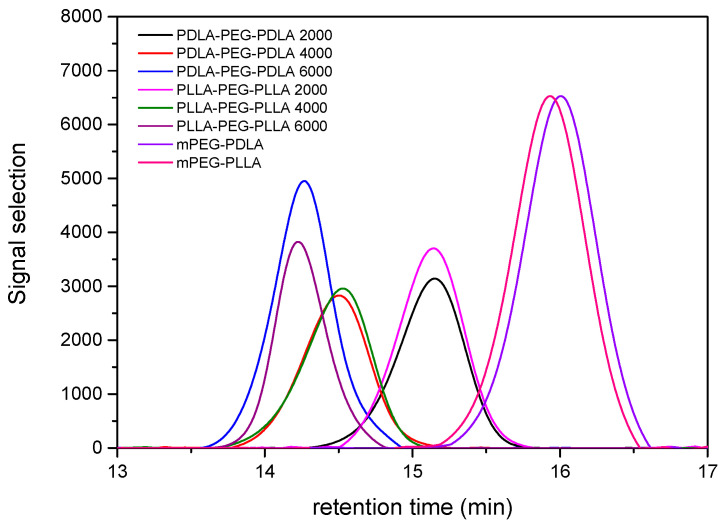
GPC chromatograms of the synthesized PLA-PEG-PLA and mPEG-PLA copolymers.

The molecular weight characteristics and polydispersity indices (PDI) of the synthesized block copolymers were determined by Gel Permeation Chromatography (GPC), and the results are summarized in Table 1 and Figure 1. A series of ABA-type triblock copolymers, where the A blocks correspond to either poly(D-lactide) (PDLA) or poly(L-lactide) (PLLA) and the central B block to poly(ethylene glycol) (PEG), were analyzed. The PEG molecular weight was varied (Mn = 2000, 4000, 6000 g/mol), while the PLA blocks were synthesized using either D- or L-lactide monomers to investigate stereochemical effects. PEG molecular weights from 1000 to 6000 g/mol were selected to cover the commonly reported range for thermoresponsive hydrogels, while PLA blocks were synthesized to ~2–6 kDa. Increasing PEG length was expected to enhance hydrophilicity and lower crystallinity, while PLA block length influences hydrophobic core formation, micelle stability, and potential drug–polymer interactions. GPC traces exhibit unimodal and symmetric distributions (Figure 1), indicating successful polymerization. The retention times shift progressively toward lower values with increasing molecular weight, consistent with the expected size exclusion behavior of the GPC column. Notably, the elution profiles of PDLA-PEG-PDLA and PLLA-PEG-PLLA samples of corresponding PEG block length are closely aligned, indicating that their chain sizes are nearly the same, regardless of the lactide stereochemistry. The number-average molecular weights (Mn) increased with PEG length, ranging from approximately 6250 to 17,800 g/mol for the triblock copolymers. The polydispersity indices (PDIs) remained relatively low in all cases (1.03–1.06), reflecting controlled polymerization and narrow molecular weight distributions. The reference samples, mPEG–PDLA and mPEG–PLLA diblock copolymers, exhibited lower molecular weights (Mn ≈ 2200–2500 g/mol) and slightly higher PDIs (1.08 and 1.10).

**Figure 2:**
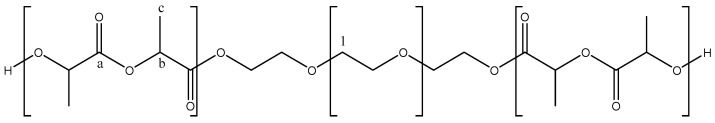
Schematic illustration of the ABA-type architecture PLA-PEG-PLA triblock copolymer.

NMR was employed herein to confirm the successful synthesis of the PLA-PEG-PLA copolymers, providing structural information on the PLA and PEG segments by assigning chemical shifts to specific protons and carbons. ^1^H NMR and ^13^C NMR spectroscopy were initially performed on both PLA and PEG in order to identify their characteristic peaks. The expected ABA-type structure is depicted in Figure 2.

**Figure 3:**
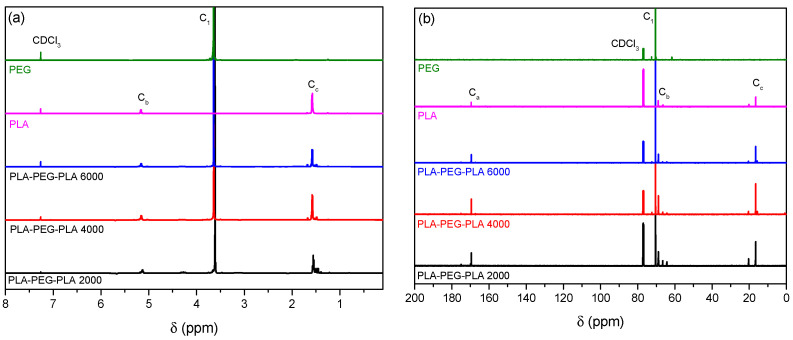
NMR spectra of the PLA-PEG-PLA triblock copolymer: (**a**) ^1^H NMR and (**b**) ^13^C NMR.

For pristine PLA, the ^1^H-NMR spectrum (Figure 3a) displays two characteristic signals at 5.2 ppm and 1.8 ppm, corresponding to the protons Hb and Hc, respectively. In the ^13^C-NMR spectrum (Figure 3b), PLA shows distinct peaks at 170 ppm, 68 ppm, and 18 ppm. The peak at 170 ppm corresponds to the carbonyl carbon (Ca) of the ester group (C=O), the peak at 68 ppm is attributed to the methine carbon (Cb) bonded to the oxygen in the CH–O group, and the signal at 18 ppm corresponds to the methyl group CH_3_ (Cc). In the PLA–PEG–PLA copolymers, a characteristic peak is observed at 3.6 ppm, assigned to the –CH_2_–O of PEG (C1), and peaks at 5.1 ppm and 1.5 ppm correspond to the CH–O (Cb) and CH_3_ (Cc) protons of the PLA, respectively. The ^13^C-NMR spectrum also displays PLA characteristic peaks at 169 ppm (Ca) C=O, 69 ppm (Cb) C–O, and 17 ppm (Cc) CH_3_.

**Figure 4:**
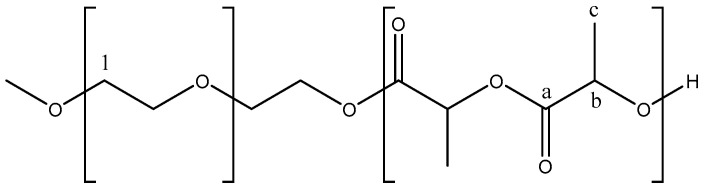
Schematic representation of the AB-type architecture, with mPEG as the terminal hydrophilic block and PLA as the hydrophobic segment.

**Figure 5:**
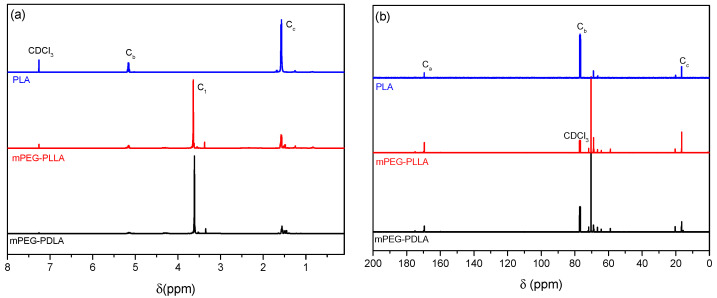
NMR spectra of the mPEG-PLA diblock copolymer: (**a**) ^1^H NMR and (**b**) ^13^C NMR.

Similarly, in the mPEG–PLA (Figure 4) copolymers, the ^1^H-NMR (Figure 5a) spectrum shows a small peak at 3.3 ppm, attributed to the methoxy group located at the terminal end of the mPEG chain. A second peak at 3.5 ppm corresponds to the etheric carbon A1 of the PEG segment. Additionally, two peaks at 5.1 ppm and 1.5 ppm are observed, which correspond to the C–O group (Cb) and the methyl group (Cc) of the PLA segment, respectively. In the ^13^C-NMR spectrum (Figure 5b), the characteristic peaks of PLA are again detected, at 169 ppm for the C=O (Ca), 69 ppm for C–O (Cb), and 17 ppm for the CH_3_ group (Cc) [16].

**Figure 6:**
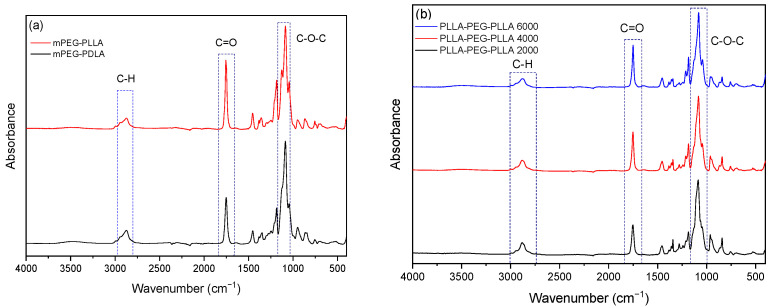
ATR–FTIR spectra of (**a**) mPEG-PLA and (**b**) PLA-PEG-PLA copolymers.

To confirm the structure of the copolymers, ATR–FTIR analysis was performed, and the results are presented in Figure 6. ATR–FTIR revealed the characteristic vibrational modes of ester and ether groups, thus confirming the successful copolymerization. For PLA, a distinct absorption band is observed at approximately 3000 cm^−1^, corresponding to C–H stretching vibrations. A sharp peak at 1744 cm^−1^ is attributed to the carbonyl stretching vibration (C=O), which is characteristic of ester groups. At 1455 cm^−1^, absorptions related to the bending vibrations of –CH_2_ and –CH_3_ groups are detected. Finally, a strong absorption band around 1080 cm^−1^ corresponds to the C–O stretching of the ester bond in the PLA structure [17].

During the analysis of mPEG–PLA copolymers (Figure 6a), absorption bands appear in the region of 3000–2800 cm^−1^, corresponding to C–H stretching vibrations. Additionally, a strong peak around 1750 cm^−1^ is associated with the carbonyl (C=O) stretching of the ester group, a characteristic feature of PLA. In both samples, a distinct absorption in the region of 1200–1000 cm^−1^ is attributed to the ether bond (C–O–C) of PEG.

During the analysis of the PLLA–PEG–PLLA and PDLA–PEG–PDLA triblock copolymers (Figure 6b), no differences were observed between the two stereoisomers. A characteristic absorption band in the region of 3000–2800 cm^−1^ corresponds to C–H stretching vibrations. Additionally, a distinct peak around 1750 cm^−1^ is associated with the carbonyl (C=O) stretching of the polyester groups in the PLA segments. Finally, in the region of 1200–1000 cm^−1^, absorption bands are observed that correspond to the ether bonds (C–O–C) of the PEG component. The diffraction peaks correspond to PLA and PEG crystallinity, in agreement with earlier reports [18,19].

### 2.2. Glass Transition and Crystallization

#### 2.2.1. XRD

**Figure 7:**
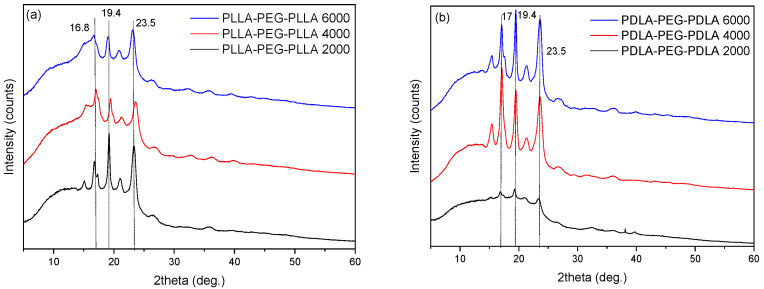
XRD patterns of (**a**) PLLA-PEG-PLLA and (**b**) PDLA-PEG-PDLA triblock copolymers.

Upon analysis of the XRD results (Figure 7), in the samples as received, the polymers were found to exhibit a semicrystalline state. Specifically, two characteristic peaks were observed at 2θ = 17° and 2θ = 19.4°, which are attributed to PLA. The presence of PEG is indicated by the peak at 2θ = 19.4°, which overlaps with that of PLA, as well as by the peak at 2θ = 23.5° [13]. An increase in the molecular weight of PEG (from 2000 to 6000) was found to influence the crystallinity of the copolymers. Specifically, higher PEG content led to a noticeable decrease in the intensity of the PLA-associated peaks, suggesting a reduction in overall crystallinity. This behavior is attributed to the flexible PEG chains disrupting the regular packing of PLA segments, thus hindering crystal formation. Furthermore, a comparison between PLLA-PEG and PDLA-PEG systems revealed similar diffraction patterns, with overlapping peak positions indicating comparable crystalline structures. On the other hand, the increase in PEG molecular weight in PDLA-PEG systems revealed an increase in the associated peaks. These subtle differences in peak sharpness and intensity suggest that the stereochemistry of the PLA segment (L- or D-form) may slightly affect the degree of crystallinity.

#### 2.2.2. DSC

**Figure 8:**
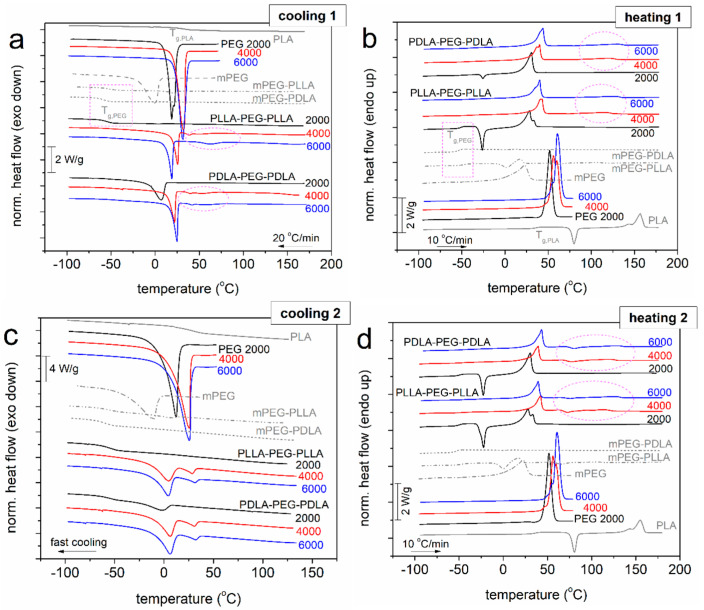
Comparative DSC traces during (**a**,**b**) cooling at 20 °C/min and heating at 10 °C/min of scan 1 and (**c**,**d**) fast cooling and heating at 10 °C/min of scan 2. The recorded heat flow is shown upon normalization to the sample mass. The circle-marked areas correspond to thermal events arising from PLA reach areas, whereas the rectangular-marked areas show the glass transition regions originating from PEG (whenever recorded).

The thermal behavior of various PEG-based copolymers and PLA-containing materials was examined via DSC under slow (Figure 8a, cooling 1) and fast (Figure 8c, cooling 2) cooling conditions, and the results are presented in Figure 8. Therein, comparing the thermal response of neat PLA and neat PEGs with the copolymers, we may conclude that the low temperature side of the DSC traces is dominated by the crystallization/melting of PEG, most probably the PEG-rich domains.

**Figure 9:**
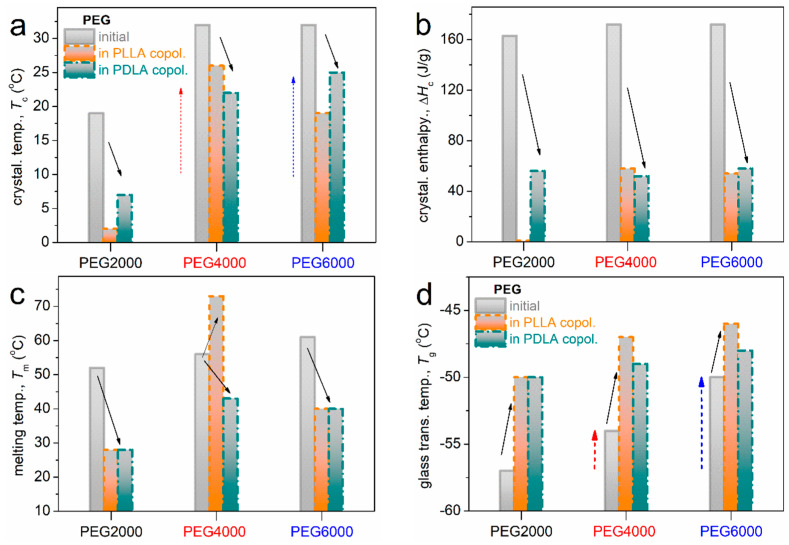
Values of interest by DSC, namely, referring to the effects imposed by the alternation in the molecular weight of PEG, in bulk and in the PLLA- and PDLA-based copolymers, and on (**a**) the crystallization temperature, (**b**) the crystallization enthalpy change, (**c**) the melting temperature, and (**d**) the glass transition temperature.

Overall, the estimated values based on the recorded thermal transitions and thermal parameters, namely, crystallization temperature (*T*_c_), crystallization enthalpy change (Δ*H*_c_), melting temperature (*T*_m_), and glass transition temperature (*T*_g_), can be seen in Figure 9a–d. Regarding neat PEGs, the increase in the molecular weight of PEG and MW_PEG_ from 2000 to 6000 g/mol leads gradually to higher crystallization temperatures (*T*_c_), melting enthalpies (Δ*H*_m_), and melting points (*T*_m_). This indicates increased crystallinity due to the ability of longer PEG chains to form ordered domains, as previously reported [20].

Coming to the copolymers, the crystallization in the copolymers is recorded between 2 and 25 °C, and, in general, seems to be mainly independent of the stereoisomeric type of lactide, but it depends on the length of PEG (MW_PEG_). In Figure 9a, *T*_c_ increases with increasing MW_PEG_, suggesting elevated nucleation ability [21]. The nucleability of PEG is hindered in the copolymers (*T*_c_ drop). The same happens in Δ_Hc_, which reflects the strong suppression of the PEG’s degree of crystallinity in the copolymers.

Notably, during the fast cooling (scan 2), the crystallization of PEG was suppressed, as compared to scan 1; nevertheless, it was not possible to eliminate it. The corresponding melting of crystals is recorded within the range from 28 to 73 °C. For the lower MW_PEG_ in the copolymers, *T_m_* in Figure 9c is lower. The effect suggests, in general, the formation of either small crystals or/and more disordered (sparser) lamellae packing. This is not the case in neat PEGs, wherein crystallization is, in general, strongly independent of MW_PEG_. Thus, we may conclude that in copolymers, the PEG regions should be “nanometric”, at least in the PEG2000-based ones, and low micrometric (a few tens of microns) for the larger MW_PEG_. This also explains the weak XRD patterns shown in the previous study [22]. The glass transition of PEG is barely recorded. In particular, it is recorded in the PEG-2000-based copolymers due to the absence of crystallization (rectangular marked areas in Figure 8). The corresponding *T_g_*s in Figure 9b, as estimated from DSC scan 2, increase in the bulk PEGs for increasing MW_PEG_, whereas they further increase in the copolymers. The PLLA blocks seem to impose slightly larger effects on the increase in *T_g_* as compared to PDLA.

Overall, these data reflect a clear interdependence of PEG and PLA segments within the copolymer architecture. This provides additional, however indirect, proof for the successful in situ copolymer synthesis.

Regarding the thermal transitions of PLA in the copolymers, we did not record the individual bulk-like behavior of PLA (gray solid lines in Figure 8). Despite that, in the PEG4000- and PEG6000-based copolymers, there seems to exist a complex temperature region (i.e., from 50 to 150 °C in Figure 8b,d), within which we identified additional weak exothermal and endothermal events (circle-marked areas). These originate from the “modified” cold crystallization and melting of PLA reach domains [23]. It is most likely that while PEG2000 precludes any significant phase separation between PEG and PLA, this is not the case for PEG4000 and PEG6000.

The introduction of PLA segments into the copolymer chain suppressed the crystallinity of PEG, particularly in the case of the diblock copolymers (mPEG-PLA), which likely results from steric hindrance and phase incompatibility between the hydrophobic PLA and hydrophilic PEG blocks that interfere with the formation of a crystalline PEG domain. The presence of two distinct melting peaks in the DSC thermograms (one attributed to PEG (~40 °C) and another to PLA (~150 °C) confirms previously proposed models involving phase separation between the two polymer components [22,24].

On the other hand, the absence of significant cold crystallization peaks in most samples indicates that crystallization occurred predominantly during synthesis or cooling, with limited reorganization upon reheating [25]. Finally, to make a comparison with similar systems, it is worth mentioning that in PLA-based block copolymers synthesized by in situ ROP, over poly(propylene adipate) blocks [26], we recorded a partial phase separation and other relevant effects, which were quite similar to the present work. Interestingly, we proved in the said previous study that the phase separation is mainly crystallization-induced [23] rather than a matter of incompatibility/selectivity between the two polymers.

### 2.3. Evaluation of Thermoresponsive Behavior and In Situ Gelation

**Figure 10:**
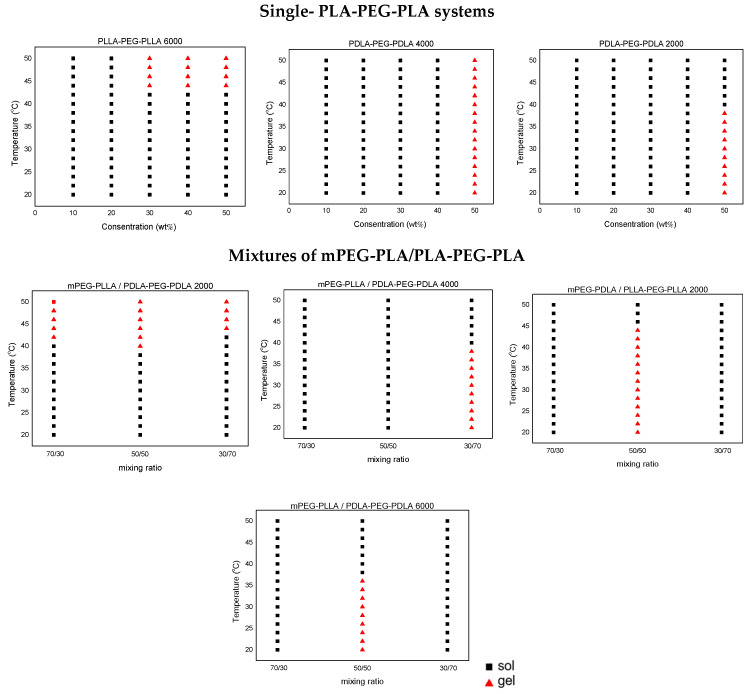
Phase diagram of the stereomixtures of micelle solutions prepared by different-sized block copolymers by the tilt method upon increase in temperature for each concentration. Plots represent solution state (black squares) and gel state (red triangles).

To evaluate the thermoresponsive behavior, all polymer samples were subjected to heating in their solution state, following the procedure outlined in the experimental section. Upon reaching the sol–gel transition temperature, the micellar dispersion transformed into a gel, a change that could be visually confirmed by inverting the vial. In stereocomplexed PLA-PEG-PLA hydrogels, this transition is believed to result from temperature-induced physical cross-linking via chain exchange between micelles [11,27,28,29,30]. The corresponding sol–gel transition temperatures for both single and mixed copolymer systems are presented in the phase diagrams shown in Figure 10.

Specifically, the following diagrams show the physical state of each polymer at temperatures ranging from 20 to 50 °C at different concentrations. Polymers that remain flowable are characterized as liquids and are depicted as black squares (sol), while those that do not flow upon inversion of the vials for at least 10 s are characterized as gels and are represented by red triangles (gel).

Regarding the polymer solutions in PBS, PLLA-PEG-PLLA 2000, PDLA-PEG-PDLA 4000, and PDLA-PEG-PDLA 6000 triblock copolymers formed in situ gels at 50 wt% concentration (Figures S1, S2 and S3, respectively). As shown in Figure 10, only samples PLLA-PEG-PLLA 2000 and PDLA-PEG-PDLA 6000 exhibited the desired thermoresponsive behavior. Notably, this was observed only at higher polymer concentrations, in contrast to more diluted solutions, which did not exhibit any significant change. On the other hand, PDLA-PEG-PDLA 4000 remained as a gel throughout the tested temperatures.

In the solutions obtained from polymer mixtures in Figure 10, a phase transition was observed both in mixtures containing L-lactide and D-lactide and in those composed exclusively of L-lactide, a phenomenon attributed to the differences in PLA stereocomplex. These blends were prepared by combining 20 wt% PBS solutions of all polymers. A comparatively low polymer concentration was chosen to ensure that the polymers remained in a liquid state before mixing. Consequently, all polymers were initially liquid. Within minutes after mixing, some polymers underwent gelation followed by reversion to the liquid phase upon temperature elevation, whereas others initially existed as liquids and transitioned to gels upon heating.

Special attention should be given to PDLA-PEG-PDLA 2000, which exhibited a gel–sol transition in PBS but switched to a sol-gel transition at all mixing ratios with mPEG-PLLA at temperatures higher than 40 °C (Figure S4). PLLA-PEG-PLLA 6000, after mixing with mPEG-PLLA at a 50/50 ratio, appeared as a gel at room temperature and converted to a liquid upon heating (gel–sol), in contrast to the initial sol–gel behavior of neat PLLA-PEG-PLLA 6000 (Figure S5). Finally, a mixture of mPEG-PDLA/PLLA-PEG-PLLA 2000 (50/50 ratio) remained as gel upon heating until 45 °C and turned into sol (gel-sol) above this temperature (Figure S6).

The rest of the samples mentioned in the experimental section did not exhibit any thermoresponsive behavior and thus were not included in the diagrams. As observed, out of the eight triblock copolymers tested, merely two—PLLA-PEG-PLLA 2000 and PDLA-PEG-PDLA 6000 at 50% *w*/*w* concentration—formed gels near physiological temperature (around 37 °C). However, the high polymer concentration required for gelation poses challenges for injectable formulations, as typical injectable hydrogels are prepared at 20–30% *w*/*w* or less to maintain acceptable viscosity and ease of syringeability. To improve practicality, strategies such as adjusting the PLA/PEG block ratio, leveraging stereocomplexation or copolymer blending, and introducing weak physical cross-links to strengthen the polymer network can be applied to lower the critical gelation concentration. These approaches will be the focus of future work to enhance the clinical applicability of these hydrogels.

### 2.4. DFO-Loaded Hydrogels

#### 2.4.1. Characterization of DFO-Loaded Hydrogels

**Figure 11:**
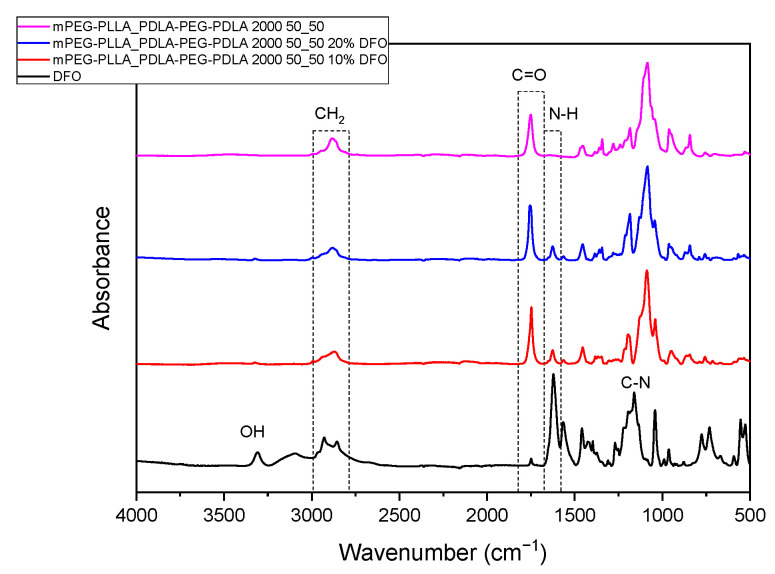
ATR–FTIR spectra of freeze-dried DFO-loaded and unloaded PLA-PEG-PLA hydrogels.

For further characterization, the hydrogels were freeze-dried. The ATR-FTIR spectra of the freeze-dried hydrogels of triblock copolymers, unloaded and loaded with deferoxamine (DFO) at 10% and 20% *w*/*w*, are presented in Figure 11. All characteristic absorption bands of both the polymer and the drug are evident, and shifts in peak positions provide insights into molecular interactions between DFO and the polymer matrix.

DFO exhibits its typical infrared absorption bands in the 1650–1600 cm^−1^ region, which are attributed to the stretching vibration of the carbonyl group (C=O) and the bending vibration of the N–H group of the primary amine. A peak at 1470 cm^−1^ corresponds to aliphatic amide C=O stretching, while the C–N stretching vibration is observed at 1200 cm^−1^. Additionally, a smaller peak around 974 cm^−1^ is assigned to N–O bending. These signals confirm the structural integrity of the drug upon incorporation into the polymer [31]. Upon DFO incorporation, notable spectral shifts suggest non-covalent interactions between the drug and the polymer. Specifically, the N-H of DFO shifts from 1622 to 1616 cm^−1^, indicating possible hydrogen bonding between the amine groups of DFO and the carbonyl of PLA. Similarly, the C–N stretching band moves from 1162 to 1187 cm^−1^, likely due to interaction between nitrile-C–H groups and the polymer. Minor shifts in the CH_2_ stretching region (from 2991 to higher wavenumbers) and the PLA carbonyl band (around 1751 cm^−1^) further support the presence of hydrogen bonding between DFO and the PLA chains. PEG, as a hydrophilic polymer and middle block in the copolymer structure, does not interact with DFO since it lacks functional groups capable of forming strong interactions.

Overall, the ATR-FTIR results confirm the successful physical incorporation of DFO into the PLA-PEG-PLA copolymer while also demonstrating specific intermolecular interactions—primarily hydrogen bonding—between drug and polymer. This type of bond plays a crucial role in controlled drug delivery by enhancing drug–polymer interactions and stabilizing the drug within the polymer matrix, reducing the initial burst release and promoting sustained drug diffusion over time. Such interactions modify the microenvironment of the drug, allowing for more controlled release profiles by slowing diffusion and degradation processes [32].

**Figure 12:**
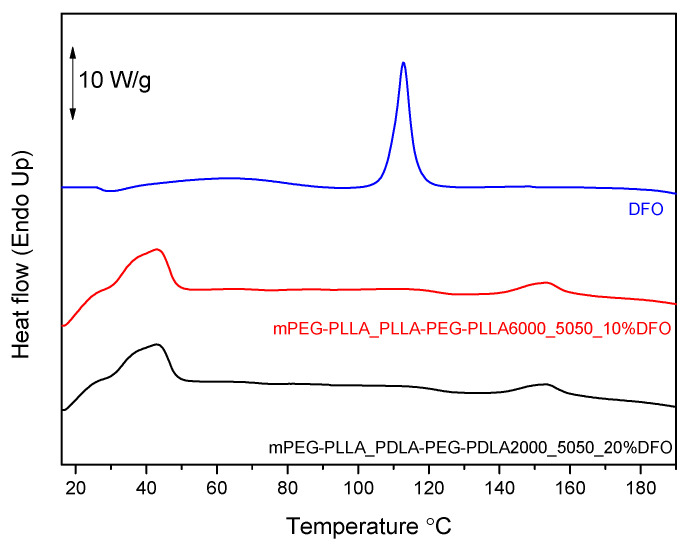
DSC thermograms of DFO-loaded PLA-PEG-PLA hydrogels.

The thermal behavior of hydrogels loaded with deferoxamine (DFO) was evaluated using differential scanning calorimetry (DSC). Pure DFO exhibited a sharp endothermic peak at approximately 113 °C, corresponding to its melting point and indicative of its crystalline nature [33]. In contrast, the DSC thermograms of the DFO-loaded PLA-PEG-PLA copolymer systems (Figure 12) showed no thermal event at this temperature, suggesting the absence of crystalline DFO in the final formulations.

Instead, two distinct endothermic transitions were observed in all samples: the first around 40 °C, corresponding to the melting point of the PEG block, and the second near 150 °C, attributed to the melting of PLA. The disappearance of the DFO melting peak in the polymeric samples indicates that the drug exists in an amorphous form.

The amorphous state of DFO is advantageous in the context of drug delivery, as it typically leads to improved solubility, enhanced bioavailability, and more predictable release kinetics compared to the crystalline form. Amorphous drugs lack long-range molecular order, which lowers the thermodynamic barrier for dissolution in physiological fluids [34]. Therefore, the DSC data confirm not only the successful incorporation of DFO into the polymer but also its physical transformation into a more pharmaceutically favorable amorphous state.

These findings support the role of PLA-PEG-PLA as an effective carrier for DFO, enabling the suppression of drug crystallization and potentially enhancing its therapeutic efficacy through improved release characteristics.

#### 2.4.2. In Vitro Drug Release

The release rate of biomolecules from polymeric systems depends on several factors, such as the crystallinity and physical state of the active substance, as well as the characteristics of the polymer carrier (e.g., molecular weight, melting point). Generally, the extent of release increases over time, depending on these parameters [35,36].

**Figure 13:**
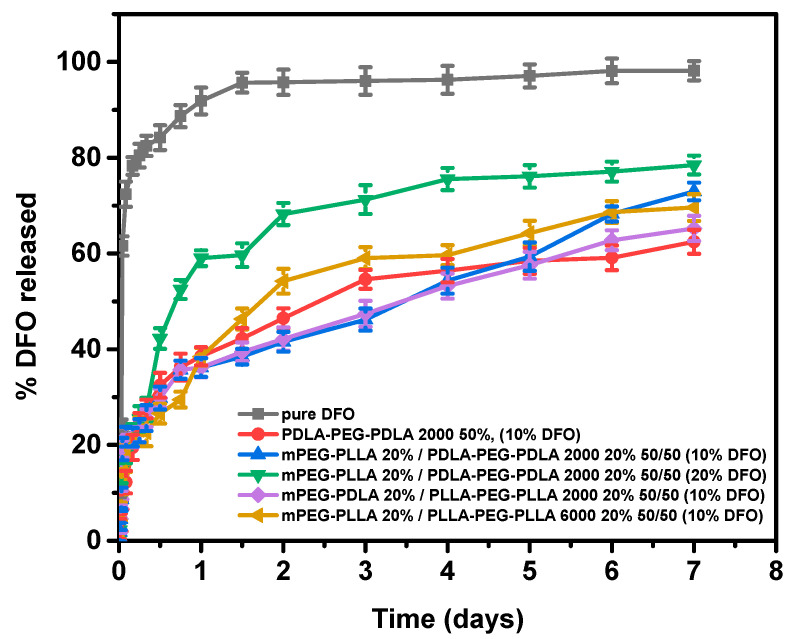
In vitro release profiles of DFO from polymeric carriers over a period of 7 days.

The dissolution rate of the polymeric systems compared to that of the pure drug over a 168 h (7-day) period is shown in Figure 13. This timeframe was selected because patients with transfusion-dependent thalassemia typically receive at least five overnight infusions with DFO per week. The in vitro release profile of deferoxamine (DFO) from the developed polymeric carriers demonstrates a biphasic pattern, typical for hydrophilic drugs. As observed in the first 12 h, a pronounced burst release occurs, likely due to the diffusion of surface drug molecules that are poorly entrapped within the polymeric network. This behavior aligns with the highly water-soluble nature of DFO and is consistent with similar release patterns reported in micellar systems. Following the burst phase, a more controlled and sustained release is observed. In clinical practice, the ideal DFO release profile would minimize the burst phase while sustaining drug levels over several days, thereby reducing the need for prolonged infusions. The initial burst observed here (>50% in 12 h) may be considered a limitation; however, it still represents a significant improvement compared to free DFO, which is released almost completely within hours. Thus, the polymeric hydrogels extend drug availability beyond free form. Future work will focus on strategies to suppress the burst through increasing the hydrophobic PLA content to slow water penetration, optimizing drug loading to minimize surface-associated drugs, and introducing weak cross-linking to reinforce the network.

Quantitative analysis over a 7-day period reveals that the cumulative drug release is strongly affected by both drug loading and polymer composition. Pure DFO exhibits nearly complete release (98.1%), as expected. Among the polymeric formulations, higher drug loading (20%) led to increased release percentages, likely due to higher concentration gradients and potential saturation effects within the carrier matrix. Furthermore, mixed systems (e.g., mPEG-PLLA/PDLA-PEG-PDLA blends) demonstrated improved release efficiency compared to single-component polymers. This suggests that copolymer composition influences the release of DFO.

Furthermore, mixed systems, particularly the mPEG–PLLA/PDLA–PEG–PDLA (50:50) blend, exhibited improved release characteristics compared to single-component polymers. This improvement can be attributed to stereocomplexation effects between L- and D-lactide segments, which reduced crystallinity (as confirmed by DSC and XRD) and generated a more homogeneous micellar network. The system displayed a biphasic profile with a moderated burst, again contrasting with the nearly immediate dissolution of free DFO, followed by sustained delivery over 7 days and achieving the highest dissolution rates among the samples.

#### 2.4.3. Biological Activity of DFO-Loaded Hydrogels

To evaluate the biological activity and iron-chelating efficiency of the DFO-loaded thermoresponsive hydrogels, the following four selected polymeric systems (Table 2) that exhibited sol-to-gel and gel-to-sol transitions without varying the molecular weight of PEG were selected and tested in HeLa cells.

**Figure 14:**
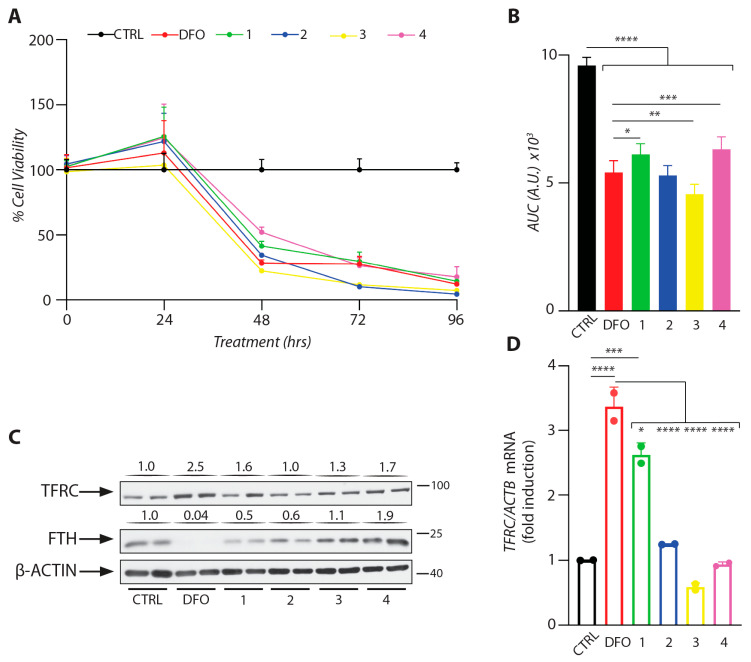
DFO-containing polymers release bioactive DFO that promotes iron deficiency responses in HeLa cells. (**A**) Kinetic analysis (0 to 96 h) of the effects of free DFO and DFO-containing polymers on the viability of HeLa cells. Cell viability was assessed by the crystal violet assay. (**B**) Area Under Curve (AUC) analysis for the cell viability curves. (**C**) Western blot analysis of TFRC, FTH, and β-ACTIN after 24 h of incubation with DFO and DFO-containing polymers. Immunoreactive bands were quantified by densitometry. Every band was normalized to the corresponding β-ACTIN band, and the fold changes compared to the control group are shown on top. (**D**) qRT-PCR analysis of *TFRC* mRNA after 24 h of incubation with DFO and DFO-containing polymers. The results were normalized to *ACTB* mRNA levels. The AUC (**B**) and qRT-PCR (**D**) data are presented as geometric mean ± geometric standard deviation; statistical analysis was performed by one-way ANOVA (* *p* ≤ 0.05; ** *p* ≤ 0.01; *** *p* ≤ 0.001; **** *p* ≤ 0.0001).

The cells were treated with the DFO-loaded hydrogels, or with free DFO or saline as controls. For all treatments, the final DFO concentration was 100 μΜ, which causes effective iron depletion [37]. Because prolonged pharmacological iron chelation eventually leads to cell death [38], we monitored cell viability over 96 h of treatment. All DFO-containing formulations induced a time-dependent decrease in cell viability, consistent with growth arrest and apoptotic cell death [38] (Figure 14A). Formulations 1 and 4 appeared somehow less toxic compared to free DFO; the reason for this is unclear. This observation is further confirmed by Area Under Curve (AUC) analysis, which revealed that these formulations resulted in higher cell viability across the tested timeframe compared to DFO-only treatment (Figure 14B).

Homeostatic adaptation to cellular iron perturbations is mediated by the IRE/IRP regulatory system. In iron-deficient cells, iron regulatory proteins (IRPs) bind to iron-responsive elements (IREs) within the untranslated regions (UTRs) of several mRNAs and control their stability or translation [39]. The binding of IRPs stabilizes transferrin receptor 1 (*TFRC*) mRNA and inhibits translation of ferritin (*FTH*) mRNA to increase iron uptake and inhibit iron storage, respectively. Thus, *TFRC* and FTH are well-established markers for cellular iron status. We analyzed TFRC and FTH expression (by Western blotting), as well as *TFRC* mRNA levels (by qRT-PCR) after a 24 h treatment of cells with the DFO formulations. Formulation 1 consistently induced *TFRC* mRNA and TFRC protein expression and suppressed FTH steady-state levels, similar to free DFO (Figure 14C,D). The other formulations appeared to mostly affect TFRC but not FTH; this may be related to their relatively reduced capacity to release DFO (Figure 13).

Overall, our experimental data suggest that DFO released from the thermoresponsive polymeric carriers, especially mPEG-PLLA/PDLA-PEG-PDLA 2000, retains its biological activity and induces expected iron deficiency responses at both protein and gene expression levels, at least in HeLa cells. These findings require corroboration in additional cell culture models (for instance, macrophages and hepatoma cells) and eventually in animal models of iron overload.

## 3. Conclusions

In this study, thermoresponsive PLA-PEG-based hydrogels were successfully synthesized and characterized as potential thermosensitive systems for controlled release of deferoxamine (DFO), an iron-chelating drug widely used in thalassemia treatment.

Spectroscopic (NMR, FTIR) and thermal (DSC) analyses confirmed the formation of both diblock and triblock copolymers, revealing the characteristic peaks of PLA and PEG segments. The most pronounced effects were observed on the crystallization and melting behavior of PEG, primarily influenced by its molecular weight, with modest contributions from D- and L-based PLA units. These interdependent structural features provided strong evidence of successful in situ copolymerization. The copolymers exhibited thermoresponsive gelation at physiologically relevant temperatures (37 °C), supporting their suitability for injectable drug delivery formulations.

DFO loading efficiency increased proportionally with drug concentration, and release studies revealed a biphasic profile characterized by an initial burst followed by a sustained release phase extending over 7 days. Among all formulations, the copolymer blend of mPEG–PLLA/PDLA–PEG–PDLA 2000 (50:50) with 20% DFO (formulation 1) showed the most favorable release characteristics, such as sol–gel transition near 37 °C, favorable biphasic release, and preserved bioactivity of released DFO in cell assays.

Importantly, we also evaluated the bioactivity of the released DFO on human HeLa cervical carcinoma cells. DFO released from thermosensitive hydrogels, and particularly from formulation 1, elicited short-term homeostatic responses and long-term cytotoxic effects comparable to those of free DFO. Treated cells showed similar or improved viability profiles relative to free DFO exposure, along with appropriate upregulation of TFRC and suppression of FTH, confirming the preservation of DFO bioactivity upon release from the hydrogels.

Taken together, these results highlight the potential of thermoresponsive, biodegradable hydrogels as delivery platforms for hydrophilic macromolecules such as DFO. However, in vivo comprehensive pharmacokinetic studies in animal models are needed before progressing toward clinical development.

## 4. Materials and Methods

### 4.1. Materials

Poly(ethylene glycol) (PEG) was used as the starting block for the synthesis of the copolymers. PEG samples of varying molecular weights (1000, 1500, 2000, 4000, and 6000 g/mol), as well as monomethoxy polyethylene glycol (mPEG, 750 g/mol), were purchased from Aldrich Co. (London, UK). Stannous octoate, an essential catalyst for the polymerization process, was also obtained from the same supplier. High-purity L-lactide (99.9%) was sourced from PURAC Biochem BV (Gorinchem, The Netherlands) under the trade name PURASORB^®^L, while high-purity D-lactide was provided by Fluorochem (Hadfield, UK). Toluene (99.8% purity) was supplied by Nv Chem-Lab (Brussels, Belgium). The hydrophilic hexadentate iron chelator Deferoxamine mesylate (Desferal^®^, MW 657.8 g/mol) was obtained from Novartis (Basel, Switzerland).

### 4.2. Synthesis of PLA-PEG-PLA Triblock and mPEG-PLA Diblock Copolymers

The ABA-type triblock copolymers of PLLA-PEG-PLLA and PDLA-PEG-PDLA were prepared by the ring-opening polymerization (ROP) of L-lactide and D-lactide, respectively, in the presence of PEG with varying PEG molecular weights (1000–6000 g/mol), employing Sn(Oct)_2_ as catalyst [11]. PEG (5 g, 2.5 mmol) and L- or D-lactide (4 g, 28 mmol) were placed in a reaction flask, equipped with a reflux condenser, a stir bar, and an addition funnel. Sn(Oct)_2_; was employed as the catalyst, and a 0.1 g/mL toluene solution of the compound (0.1 g, 0.25 mmol) was subsequently added. The reaction flask was sealed and evacuated to remove moisture and then converted to N_2_ gas. The reaction temperature was 130 °C and was allowed to react overnight. The reaction mixture was cooled to room temperature and solidified. Diblock copolymers (mPEG–PLA) were also synthesized via ROP of L- or D-lactide (7.2 g), mPEG (10 g), and Sn(Oct)_2_ (0.2 mL) as the catalyst at a 0.1 g/mL toluene solution. Catalyst concentration was ≈0.5 mol% relative to lactide and ≈1 mol% relative to the PEG initiator, expressed as the molar ratio of Sn(Oct)_2_ to each compound. Crude polymers were purified by dissolution in chloroform and precipitation in cold diethyl ether to remove residual monomers and catalyst traces. Reaction yields reached up to 80–90%.

### 4.3. Characterization of the Synthesized Copolymers

#### 4.3.1. Nuclear Magnetic Resonance (^1^H NMR)

The NMR spectra of the prepared copolymers were recorded using an Agilent spectrometer (Santa Clara, CA, USA) operating at 500 MHz for proton analysis under room temperature conditions. For sample preparation, deuterated chloroform (CDCl_3_) was used as solvent, forming 5% *w*/*v* solutions. The acquisition involved 32 scans for ^1^H NMR and 512 scans for ^13^C NMR. Additionally, a uniform sweep width of 6 kHz was consistently applied in all measurements.

#### 4.3.2. Fourier-Transform Infrared Spectroscopy (ATR–FTIR)

ATR-FTIR spectra for all samples were acquired using a Shimadzu IRTracer-100 spectrophotometer (Kyoto, Japan) equipped with a QATR™ 10 Single-Reflection ATR accessory featuring a diamond crystal. Measurements were conducted in absorbance mode within the spectral range of 450–4000 cm^−1^ at a resolution of 2 cm^−1^. Each spectrum was produced by averaging 32 scans. Prior to analysis, the spectra underwent baseline correction and normalization.

#### 4.3.3. Gel Permeation Chromatography (GPC)

The molecular weights of the samples were assessed via Gel Permeation Chromatography/Size Exclusion Chromatography (GPC/SEC). Analysis was carried out using an Agilent 1260 Infinity II LC system (Agilent Technologies, Santa Clara, CA, USA) equipped with an isocratic G7110B pump, a G7129A autosampler, a Refractive Index Detector (RID) G7162A, and a PLgel 5 µm (50 × 7.5 mm) guard column in series with two PLgel 5 µm (300 × 7.5 mm) MIXED-C columns. For system calibration, three poly(methyl methacrylate) (PMMA) standards with molecular weights ranging from 0.535 to 1591 kg/mol were utilized. Sample solutions were prepared at a concentration of 3 mg/mL and filtered through PTFE membranes with a pore size of 0.45 µm. Each run involved an injection volume of 20 μL and a total analysis time of 30 min. Both the columns and the RID were maintained at a constant temperature of 40 °C.

#### 4.3.4. Differential Scanning Calorimetry (DSC)

Calorimetric analysis was conducted using a TA Q200 differential scanning calorimeter (TA Instruments, New Castle, DE, USA), which had been calibrated for heat capacity using sapphire standards and for temperature and enthalpy using indium. Approximately 8 mg of each sample was sealed in aluminum Tzero Hermetic pans (TA Instruments) and analyzed over a temperature range of −100 to 180 °C at a fixed heating rate of 10 °C/min. Upon a first heating, performed to erase the thermal history and maximize the sample-pan thermal contact, two cooling–heating scans were performed (scans 1 and 2 in the following). In scan 1, the cooling rate of 20 °C/min, whereas in scan 2, the samples were cooled at the fastest cooling rate [non-linear T(t), jump command in TA Universal Analysis 2000 software (version 4.5A)TA software]. More specifically, the non-linear rate here involves higher temperatures, e.g., at the expected crystallization region of PLA, a cooling rate estimated at about 90–100 °C/min, and at lower temperatures, e.g., at the expected crystallization region of PEG and mPEG, a cooling rate estimated at about 60–70 °C/min. Within the very low temperatures, the cooling rate is lower. The latter aimed at the suppression of crystallization(s). All measurements were carried out under a high-purity nitrogen atmosphere (99.9995%).

#### 4.3.5. X-Ray Diffraction (XRD)

X-ray diffraction (XRD) measurements were conducted at room temperature across a 2θ range of 5° to 50°, using a scanning rate of 1°/min. The analysis was performed with a MiniFlex II diffractometer (Chalgrove, Oxford, UK), employing Cu Ka radiation (λ = 0.154 nm) for crystalline phase identification.

### 4.4. In Situ Gelation and Investigation of Thermoresponsive Behavior

As the polymers used in this study were initially in solid form, they were dissolved in phosphate-buffered saline (PBS) to produce aqueous solutions; PBS (0.01 M) was prepared using ultrapure water and 0.137 M NaCl, 0.0027 M KCl, while its pH was adjusted to 7.4. PLLA-PEG-PLLA and PDLA-PEG-PDLA solutions at concentrations of 10–50% *w*/*w,* which were prepared in PBS to examine the gelation behavior at 20 °C, as shown in Table 3. PLA−mPEG and PLA−PEG−PLA copolymers were separately dissolved in PBS and stirred vigorously with a magnet at room temperature until completely dissolved to prepare copolymer solutions (20 wt%). The solutions were then kept at +4 °C for 24 h for equilibration before mixing. The solutions of mPEG-PLA and PLA−PEG−PLA were mixed at 600 rpm for 1 h at RT until complete dissolution at different predetermined mass (70:30, 50:50, and 30:70) ratios to examine the in situ gelation behavior at 20 °C, as shown in Table 4. Polymer solutions or polymer blends were further investigated for sol–gel or gel–sol transitions. Prior to investigation, each solution was equilibrated in a refrigerator at 4 °C for 24 h. Solutions were placed in vials, sealed with a screw cap to prevent PBS evaporation, and transferred to a temperature-controlled water bath, in which the temperature gradually increased from 20 to 50 °C with a 2 min hold at each temperature. The physical state of the mixture was reported at each temperature interval by tilting the vial. The sol–gel temperature was determined using the tube inversion technique to identify the phase transition point. If the mixture flowed, then it was reported as a solution, and if it did not flow (the sample remained adhered to the bottom of the inverted tube) for at least 10 s, it was reported as a gel [40]. The transition temperature was recorded for each sample, and the results were plotted as phase diagrams to display the sol–gel transition temperatures.

### 4.5. High-Pressure Liquid Chromatography (HPLC), Quantitative Analysis, and Drug Loading

Quantitative analysis and drug loading were carried out using a Shimadzu HPLC system (Kyoto, Japan), which included a degasser (DGU-20A5), a liquid chromatograph (LC-20 AD), an autosampler (SIL-20AC), a UV/Vis detector (SPD-20A), and a column oven (CTO-20AC). A CNW Technologies Athena C18 column (120 A, 5 µm, 250 mm × 4.6 mm) was used, with a column temperature set to 25 °C. The mobile phase consisted of a mixture of acetonitrile (ACN) and ultrapure water (H_2_O) in a ratio of 20/80 (*v*/*v*). The flow rate was maintained at 0.8 mL/min, and the DFO concentration was determined using an (Shimadzu, Kyoto, Japan) HPLC-UV detector at 218 nm, based on a previously established calibration curve. The flow rate was set at 1.2 mL/min, and the injection volume was 20 μL. The calibration curve was generated by diluting a 1 mg/mL DFO stock solution in methanol to concentrations of 0.01, 0.02, 0.05, 0.25, 0.5, 0.75, 1, 2.5, 5, 10, 20, and 50 ppm using the mobile phase. The drug loading (DL) was then calculated using the following equation:

(1) $$Drug\;loading=\frac{W_{experimental}}{W_{theoretical}}\times 100$$

### 4.6. In Vitro Evaluation of Cell Viability

#### 4.6.1. Cell Culture

HeLa cervical carcinoma cells were grown under normal conditions (37 °C, 5% CO_2_) in a humidified incubator. Cells were cultured in Dulbecco’s modified Eagle medium (DMEM, Multicell, Woonsocket, RI, USA; Cat. No: 319-005-CL) supplemented with 10% Fetal Bovine Serum (Multicell, Cat. No: 080150) and 100 U/mL penicillin/streptomycin (Multicell, Cat. No: 450-201-EL). All the treatment media contained 100 μM of DFO (for the polymer-containing solutions, the DFO concentration was calculated assuming 100% release efficiency). For qRT-PCR and Western blotting analyses, the cells were harvested, washed in cold PBS, and separated into two fractions (one for qRT-PCR and one for Western blotting). For every condition (control and treated groups), two biological replicates were used; qRT-PCR assays were performed with two technical replicates per sample.

#### 4.6.2. Quantitative Real-Time PCR (qRT-PCR)

Total RNA was isolated from the cell lysates using the RNeasy Mini kit (Qiagen, Hilden, Germany; Cat. No: 74106) according to the manufacturer’s instructions. RNA concentration and purity were assessed by 230/260/280 nm absorbance using NanoDrop (Thermo Fisher Scientific, Waltham, MA, USA). qRT-PCR was performed using gene-specific primers (Table 5), and the data were normalized to *ACTB*. The relative RNA expression was calculated by the comparative Ct method, and the results are presented as fold changes compared to untreated cells (control).

#### 4.6.3. Western Blot

The cell pellets were lysed in lysing buffer (50 mM Tris–HCl, 15 mM NaCl, 0.5% *w*/*v* sodium deoxycholate, 0.1% SDS, and 0.1% Triton-X-100) containing protease inhibitors (Roche, Basel, Switzerland; Cat. No: 11836153001). Protein samples containing 20 μg of total protein were analyzed by SDS-PAGE on a 12% SDS-PAGE gel, and they were transferred onto a nitrocellulose membrane (BioRad, Hercules, CA, USA) according to standardized procedures. A pre-stained protein ladder was used to monitor the protein separation (Thermo Fisher Scientific, Waltham, MA, USA; Cat. No:26616). The blots were blocked with 10% fat-free skim milk in TBS-T (Tris-buffered saline (TBS), 0.1% (*v*/*v*) Tween-20) and probed O/N with primary antibodies against transferrin receptor 1 (Zymed, Thermo Fisher Scientific, Waltham, MA, USA; mouse monoclonal; 1:1000 diluted), H-ferritin (Novus Biologicals, Centennial, CO, USA; rabbit polyclonal; 1:1,000 diluted), and β-actin (Sigma-Aldrich, St. Louis, MO, USA; rabbit polyclonal; 1:1,000 diluted). The next day, they were washed 3 times with TBS-T and incubated with 1:5,000-diluted anti-mouse or 1:20,000-diluted anti-rabbit peroxidase-coupled secondary antibodies for 1.5 h. All antibodies were diluted in 5% fat-free skim milk in TBS-T. Immunoreactive bands were detected by enhanced chemiluminescence with the ECL Western Blotting Detection Reagent (Cytiva, Marlborough, MA, USA; Cat. No: RPN2209).

#### 4.6.4. Crystal Violet Cell Viability Assay

The cells were seeded in 96-well tissue culture plates (5,000 cells/well) and left to attach for 18 h in full DMEM. Eight biological replicates were measured for every condition/timepoint (control and treated groups). Then (timepoint “0”), the old medium was removed and fresh treatment medium containing 100 μM of DFO was added. The cell viability was assessed every 24 h for the next 96 h by the crystal violet assay [41]. In brief, at every timepoint, the cells were washed twice with cold PBS and fixed with 100 μL/well ice-cold methanol for 15 min. Then, they were washed once with cold PBS and incubated for 30 min with 100 μL/well 0.5% crystal violet in 2% ethanol. The dye was removed completely by multiple gentle washes with tap water, and the wells were left to air-dry completely. When dry, 100 μL/well of 2% SDS was added, and the cells were solubilized for 30 min on a shaker. Then, the absorbance was measured at 590 nm using a microplate reader, and the results are presented as % percentage of untreated cells (control).

#### 4.6.5. Statistical Analysis

GraphPad Prism software (v10.5.0, GraphPad, San Diego, CA, USA) was used for statistical analysis by one-way ANOVA with Bonferroni post hoc test comparison. A probability value of *p* < 0.05 was considered statistically significant.

## Figures and Tables

**Table 1:** Copolymers’ molecular weights and PDIs as determined by GPC/SEC.

Block Copolymer	Mn (g/mol)	PDI
PDLA-PEG-PDLA 2000	6251	1.06
PDLA-PEG-PDLA 4000	13,374	1.05
PDLA-PEG-PDLA 6000	17,343	1.04
PLLA-PEG-PLLA 2000	6266	1.05
PLLA-PEG-PLLA 4000	12,736	1.05
PLLA-PEG-PLLA 6000	17,817	1.03
mPEG-PDLA	2209	1.08
mPEG-PLLA	2530	1.10

**Table 2:** Selected diblock and triblock mPEG-PLA (mPEG 750 g/mol) and PLA-PEG-PLA (PEG 2000 g/mol) copolymer hydrogels loaded with DFO that were evaluated for biological activity and iron-chelating efficiency.

Formulation	Sample	DFO (mg)	PBS (% *w*/*v*)	Mass Ratio
1	mPEG-PLLA 750/PDLA-PEG-PDLA 2000	2	20%	50/50
			20%	
2	mPEG-PDLA 750/PLLA-PEG-PLLA 2000	2	20%	50/50
			20%	
3	mPEG-PLLA 750/PLLA-PEG-PLLA 2000	2	20%	50/50
			20%	
4	PDLA-PEG-PDLA 2000	2	50%	-

**Table 3:** PBS solutions of diblock and triblock copolymers. Brackets denote the molecular weight of PEG employed in each sample.

PLLA-PEG-PLLA/PBS (% *w*/*w*)					
PLLA-PEG-PLLA (1000)	10%	20%	30%	40%	50%
PLLA-PEG-PLLA (1500)	10%	20%	30%	40%	50%
PLLA-PEG-PLLA (2000)	10%	20%	30%	40%	50%
PLLA-PEG-PLLA (4000)	10%	20%	30%	40%	50%
PLLA-PEG-PLLA (6000)	10%	20%	30%	40%	50%
PDLA-PEG-PDLA (2000)	10%	20%	30%	40%	50%
PDLA-PEG-PDLA (4000)	10%	20%	30%	40%	50%
PDLA-PEG-PDLA (6000)	10%	20%	30%	40%	50%
mPEG-PLLA	10%	20%	30%	40%	50%
mPEG-PDLA	10%	20%	30%	40%	50%

**Table 4:** PBS blends of PDLA-PEG-PDLA and PLLA-PEG-PLLA triblock copolymers with mPEG-PLA diblock copolymers. Brackets denote the molecular weight of PEG employed in each sample.

Polymer Blends (Mass Ratio 70:30, 50:50, and 30:70)
mPEG-PLLA/PDLA-PEG-PDLA (2000)
mPEG-PLLA/PDLA-PEG-PDLA (4000)
mPEG-PLLA/PDLA-PEG-PDLA (6000)
mPEG-PDLA/PLLA-PEG-PLLA (2000)
mPEG-PDLA/PLLA-PEG-PLLA (4000)
mPEG-PDLA/PLLA-PEG-PLLA (6000)
mPEG-PLLA/PLLA-PEG-PLLA (6000)

**Table 5:** List of primers used for qRT-PCR.

Gene	Forward Primer Sequence	Reverse Primer Sequence
*TFRC*	GCAAGTAGATGGCGATAACAG	GACGATCACAGCAATAGTCCC
*ACTB*	AGGATGCAGAAGGAGATCACT	GGGTGTAACGCAACTAAGTCATAG

## Data Availability

Data is contained within the article or Supplementary Materials.

## Supplementary Materials

The following supporting information can be downloaded at https://www.mdpi.com/article/10.3390/gels11090742/s1, Figure S1: Visual observation of PDLA-PEG-PDLA (PEG 2000 g/mol) triblock copolymers at various concentrations. Gel state occurred only for 50 wt% polymer concentration; Figure S2: Visual observation of PDLA-PEG-PDLA (PEG 4000 g/mol) triblock copolymers at various concentrations. Gel state occurred only for 50 wt% polymer concentration; Figure S3: Visual observation of PDLA-PEG-PDLA (PEG 6000 g/mol) triblock copolymers at various concentrations. Gel state occurred only for 50 wt% polymer concentration; Figure S4: (a) Sol state of PDLA-PEG-PDLA 2000 (20 wt%) triblock copolymer, (b) initial sol state of PDLA-PEG-PDLA 2000 triblock copolymer mixed with mPEG-PLLA and c) sol–gel transition above 40 °C of PDLA-PEG-PDLA 2000 triblock copolymer mixed with mPEG-PLLA at all mixing ratios; Figure S5: (a) Initial sol state of PLLA-PEG-PLLA 6000 20 wt% triblock copolymer at room temperature, (b) initial sol state of mPEG-PLLA 20 wt% at room temperature, (c) initial gel state of PLLA-PEG-PLLA 6000 triblock copolymer after mixing (50/50 ratio) with mPEG-PLLA at room temperature and (d) gel–sol transition upon heating; Figure S6: Gel state of mPEG-PDLA/PLLA-PEG-PLLA 2000 mixture (50/50 ratio) upon heating until 45 °C.

## Abbreviations

The following abbreviations are used in this manuscript:

PLA	Poly(Lactic Acid)
PEG	Polyethylene Glycol
DFO	Deferoxamine
DSC	Differential Scanning Calorimetry
XRD	X-Ray Diffraction
FTIR	Fourier-Transform Infrared Spectroscopy
NMR	Nuclear Magnetic Resonance
