# Supplementary material for: Amphiphilic Thermoresponsive Triblock PLA-PEG-PLA and Diblock mPEG-PLA Copolymers for Controlled Deferoxamine Delivery
Source: Gels. 2025 Sep 15;11(9):742. doi: 10.3390/gels11090742 (PMC12469683; doi:10.3390/gels11090742)
Supplement: Supplementary file 1 [file gels-11-00742-s001.zip › gels-3834031-supplementary.pdf]

Supplementary Materials

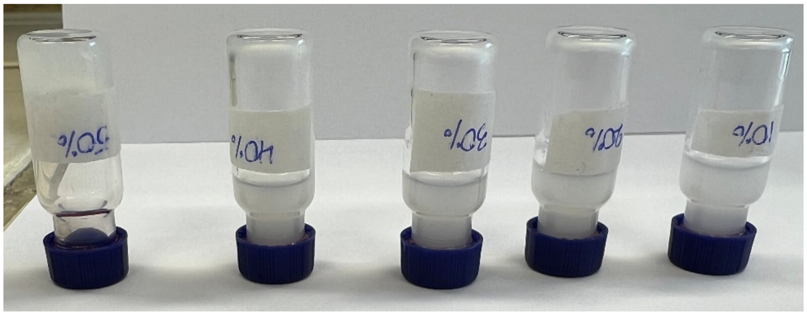

Figure S1. Visual observation of PDLA-PEG-PDLA (PEG 2000 g/mol) triblock copolymers at various concentrations. Gel state occurred only for 50 wt% polymer concentration.

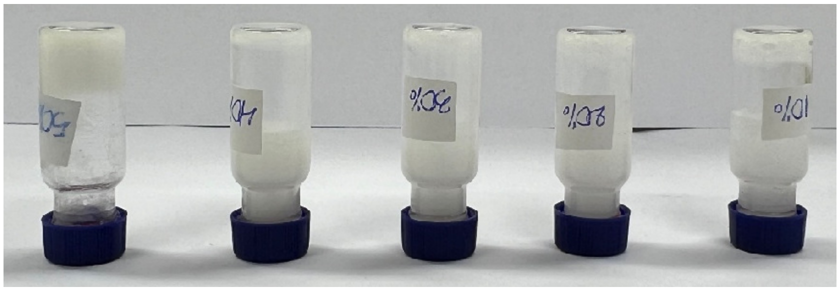

Figure S2. Visual observation of PDLA-PEG-PDLA (PEG 4000 g/mol) triblock copolymers at various concentrations. Gel state occurred only for 50 wt% polymer concentration.

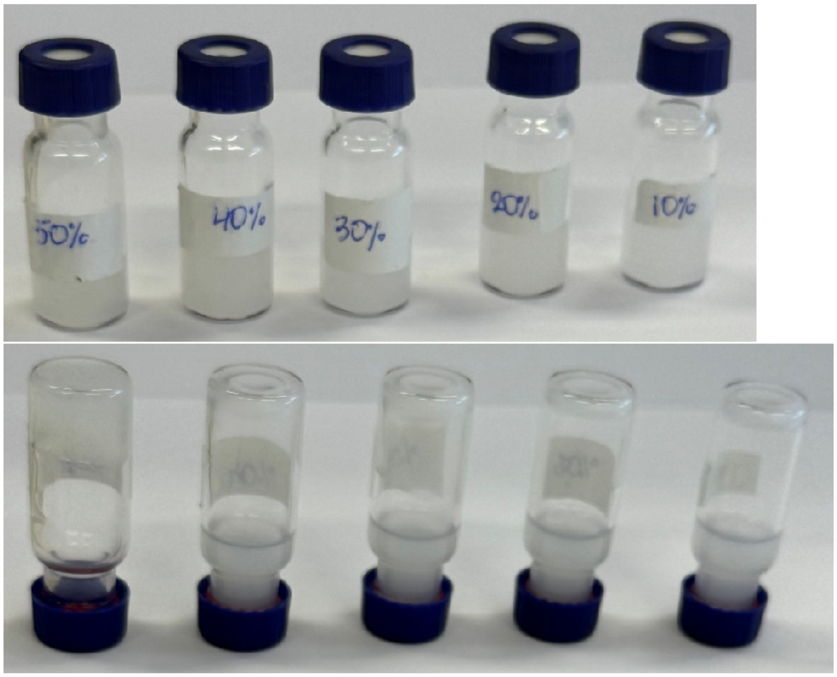

Figure S3. Visual observation of PDLA-PEG-PDLA (PEG 6000 g/mol) triblock copolymers at various concentrations. Gel state occurred only for 50 wt% polymer concentration.

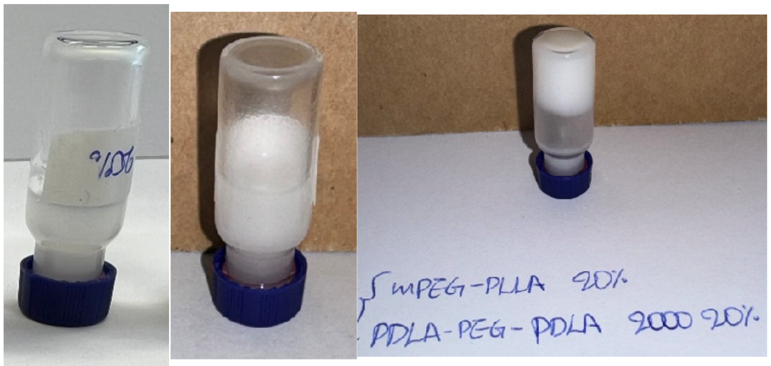

(a) (b) (c)

Figure S4. (**a**) Sol state of PDLA-PEG-PDLA 2000 (20 wt%) triblock copolymer, (**b**) initial sol state of PDLA-PEG-PDLA 2000 triblock copolymer mixed with mPEG-PLLA and (**c**) sol–gel transition above 40 °C of PDLA-PEG-PDLA 2000 triblock copolymer mixed with mPEG-PLLA at all mixing ratios.

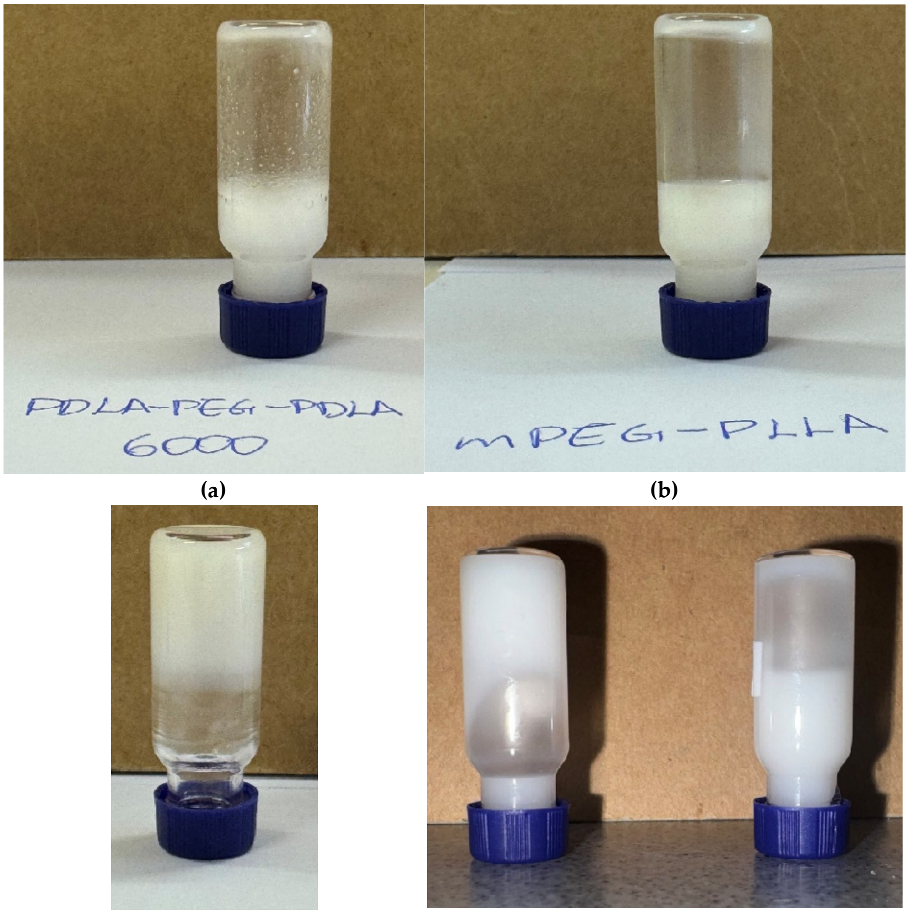

(a) (b)

(c) (d)

Figure S5. (**a**) Initial sol state of PLLA-PEG-PLLA 6000 20 wt% triblock copolymer at room temperature, (**b**) initial sol state of mPEG-PLLA 20 wt% at room temperature, **c**) initial gel state of PLLA-PEG-PLLA 6000 triblock copolymer after mixing (50/50 ratio) with mPEG-PLLA at room temperature and (**d**) gel–sol transition upon heating.

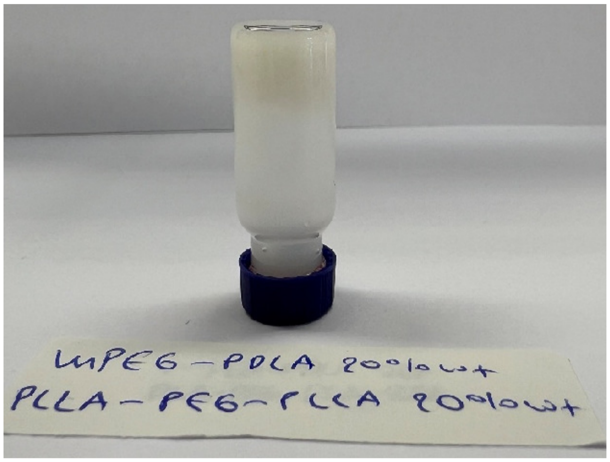

Figure S6. Gel state of mPEG-PDLA/PLLA-PEG-PLLA 2000 mixture (50/50 ratio) upon heating until 45 °C.
